# Photo-Responsive Shape-Memory and Shape-Changing Liquid-Crystal Polymer Networks

**DOI:** 10.3390/ma6010116

**Published:** 2013-01-02

**Authors:** Danish Iqbal, Muhammad Haris Samiullah

**Affiliations:** 1 Max-Planck-Institut für Eisen Forschung GmbH, Max-Planck-str.1, 40237, Düsseldorf, Germany; 2 Institute of Chemistry, Martin-Luther-Universität Halle-Wittenberg, Von-Danckelmann-Platz 4, 06120 Halle, Germany; E-Mail: muhammad.samiullah@student.uni-halle.de

**Keywords:** smart materials, shape-memory polymers, shape-changing polymers, liquid-crystalline networks, liquid-crystalline elastomers, azobenzenes

## Abstract

“Surrounding matters” is a phrase that has become more significant in recent times when discussing polymeric materials. Although regular polymers do respond to external stimuli like softening of material at higher temperatures, that response is gradual and linear in nature. Smart polymers (SPs) or stimuli-responsive polymers (SRPs) behave differently to those external stimuli, as their behavior is more rapid and nonlinear in nature and even a small magnitude of external stimulus can cause noticeable changes in their shape, size, color or conductivity. Of these SRPs, two types of SPs with the ability to actively change can be differentiated: shape-memory polymers and shape-changing polymers. The uniqueness of these materials lies not only in the fast macroscopic changes occurring in their structure but also in that some of these shape changes are reversible. This paper presents a brief review of current progress in the area of light activated shape-memory polymers and shape-changing polymers and their possible field of applications.

## 1. Smart Polymer Materials

Smart materials have revolutionized material science due to their capability of executing specific functions in response to changes in stimuli and, therefore, have potential applications in many areas; for instance, as actuators, sensors and micro-pumps. Some SPs have the ability to actively move, on applying an appropriate stimulus. The characteristic feature that actually makes them “smart” is their ability to respond in a specific way, to very slight changes in the surrounding environment, such as temperature, pH, light, magnetic or electric field, or the presence of biological molecules. The uniqueness of these materials lies not only in the fast macroscopic changes occurring in their structure but also that some of these shape changes are reversible [1,2,3,4]. Depending on their response to external stimuli, smart polymers can be further classified into two sub-groups: shape-memory polymers (SMPs) and shape-changing polymers (SCPs).

This paper presents a brief review on current progress in the area of light activated shape-memory polymers and shape-changing polymers and their possible field of applications. This review will not cover shape-memory polymers and the shape-changing polymers using stimuli other than light as they are already been a part of some excellent reviews [3,5,6,7,8,9,10,11].

### 1.1. Shape-Memory Polymers (SMPs)

**Figure 1:**
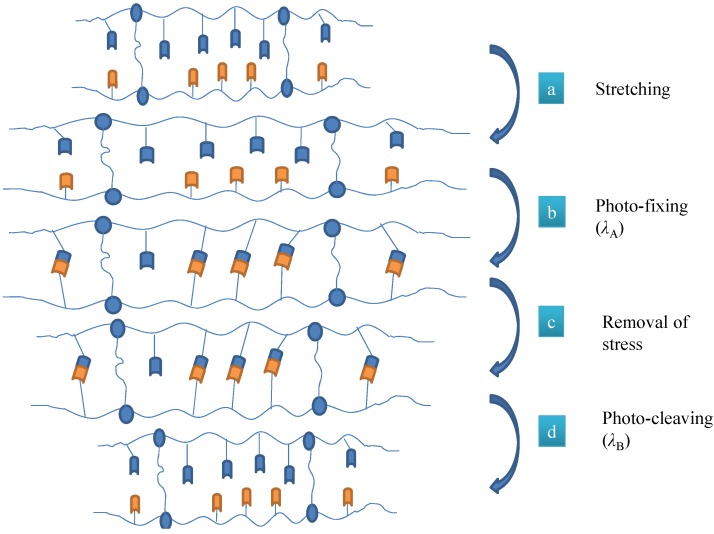
Schematic representing the molecular mechanism of photoinduced SMP [13], (**a**) stretching by applying stress, (**b**) photo-fixing by illumination with light of wavelength *λ*_A_, (**c**) removal of external stress, (**d**) photo-cleaving by exposing to light of wavelength *λ*_B_.

SMPs have the unique ability of returning to its original or permanent state after being transformed or altered by the external stimulus. This temporary shape is stable until the SMP is exposed to an appropriate stimulus such as heat or light (see Figure 1). SMP can go up to two or sometimes three shape transformations and the most popular way to achieve this is to use temperature as an external stimulus [12]. Along with the temperature change, the shape change of SMPs can also be triggered by light [13], electricity [14,15] or magnet [16]. The movement occurring during recovery is predefined as it reverses the mechanical deformation, which leads to the temporary shape.

The shape-memory effect (SME) is not an intrinsic material property but a functionalization of a material and the SME depend only combining polymers molecular architecture along with a tailored processing and programming methods. Generally, SMPs are crosslinked polymer networks equipped with suitable molecular switches, which are sensitive to external stimuli. The crosslinks can be chemical (covalent bonds) or physical in nature (intramolecular interactions).

### 1.2. Shape Changing Polymers (SCPs)

**Figure 2:**
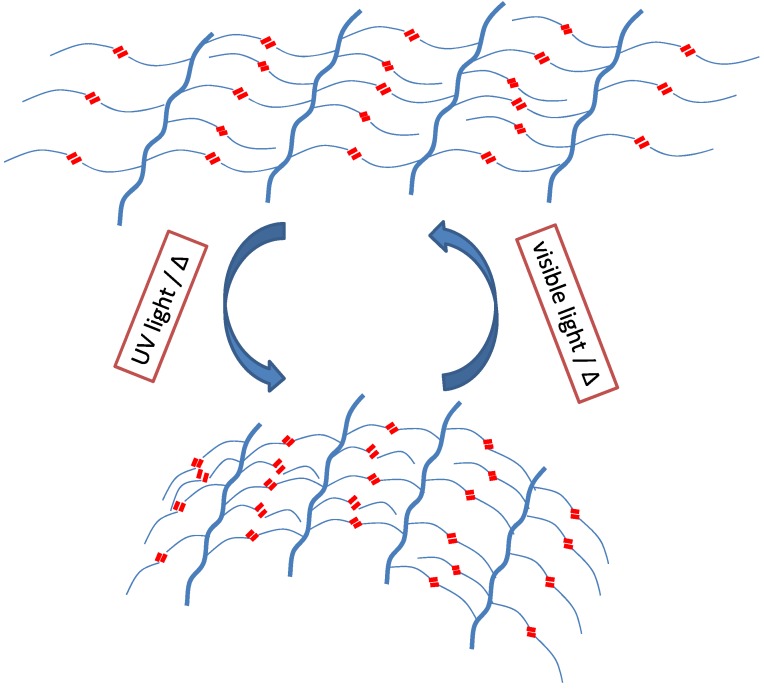
Schematic representation of photo-responsive SCP process. Polymer chains functionalized with photo-active molecules subject to shape change on exposing it to suitable wavelength of light. The original shape is recovered as soon as the stimulus is turned off.

SCPs alter their shape, e.g., shrink or bend, as long as they are exposed to an appropriate stimulus and the original shape is achieved as soon as the stimulus is terminated, demonstrated schematically in Figure 2. This shape-changing capability can be repeated several times (without applying any stress). A SCP is different from a SMP in that the geometry of the movement of the sample is determined by its original three-dimensional shape [4,17]. Most commonly used stimuli for SCPs are heat and light. Light is a particularly fascinating stimulus because it can be precisely modulated in terms of wavelength, polarization direction and intensity, allowing non-contact control. To be light responsive, polymers have to be equipped with photosensitive functional groups or fillers, e.g., cinnamic acid or azobenzenes [18]. The incorporation of such photosensitive groups or molecules into a tailored polymer surrounding in combination with functionalization process is a well-established strategy for transferring effects from the molecular level into effects that are macroscopically visible.

Photo-responsive liquid-crystalline elastomers (LCEs) are one of the interesting classes of smart materials because they combine the anisotropic aspects of LC phases and the rubber elasticity of polymer networks.

## 2. Liquid Crystalline Materials

Liquid crystals (LCs) are a unique state of matter between a solid and a liquid. The molecules in LC material typically do not exhibit any long-range positional order but they do show some extent of orientational order. The characteristic orientational order of the LC state is between the solid and liquid phases and this is the origin of the term mesogenic state, used synonymously with LC state [19].

**Figure 3:**
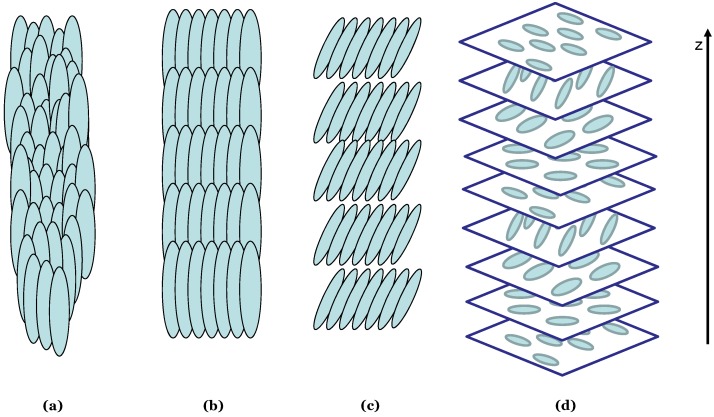
Schematic representation of different Liquid crystal (LC) phases, (**a**) nematic phase, (**b**) smectic A phase, (**c**) smectic C phase, (**d**) cholesteric phase.

LC state can be achieved in two ways *i.e.*, lyotropically, by varying a composition of a multi-component system, or thermopically, by varying temperature. A lyotropic LC phase is achieved by dissolving amphiphilic molecules in a solvent where their phase transitions can be observed through the addition or removal of solvent. Thermotropic LC structures are observed in a particular temperature range. At a high temperature, the LC material shows a typical isotropic behavior of a liquid while at too low temperatures it shows typical crystal structures of a solid. Moving from low to high temperature, the LC material exhibits various different structures [20]. The three important types of phases for calamitic LCs are nematic, smectic, and cholesteric shown schematically in Figure 3.

In the nematic phase structure, LC molecules are arranged parallel to the molecular long axis while having the freedom of rotating and moving on either direction of their long axis. This particular orientation results in making a long range orientational order but a short positional order of the LC structure. An average direction of all the molecular long axes, defines the overall directional director “z” and due to this orientational order, they show anisotropy in various properties like, optical properties (birefringence), viscosity, electrical and magnetic response, *etc.* [20]. In contrary to the nematic phase, molecules in the smectic phase found at relatively lower temperatures and possess both the orientational and positional order. They are biaxially oriented and form layers where they are not only aligned with respect to their long axis but also to one of their shorter axis. There are various types of smectic phases represented by the alphabets A, B, C, E, F, *etc.*, depending largely upon their molecular arrangement within a layer. Smectic A and smectic C phases are most common among them and differ only by the position of molecular long axis with regard to the layer’s normal axis. In smectic A structure, molecular long axis is parallel to the layers normal axis while in smectic C phase it is tilted with an angle *θ* as shown schematically in Figure 3. Other categories have usually been classified according to the crystal structure of molecules within the layer e.g., in smectic B phase, molecules are arranged in hexagonal phase centered structure while in smectic E they form a orthorhombic assembly [21].

The chiral or the cholesteric phase is similar to that of the nematic phase on a local scale. As in the nematic phase, the molecules can be described by a director; however, the director in the cholesteric phase is twisted about an axis normal to the molecular orientation, following a helical path. The distance over which the molecular director rotates for a complete 360° along the helix axis is defined as the pitch of cholesteric helix. The twist can be right-handed or left-handed depending on the molecular conformation. Iridescent colors are characteristic of cholesteric phases [22,23].

## 3. Liquid-Crystalline Elastomers (LCEs)

LCEs are unique materials that exhibit the properties of elastomers (entropic elasticity) and liquid crystals (self-organization) [24,25,26,27]. Due to the LC properties, mesogens in LCEs show an aligned and coupled crosslinked structure, which leads to many characteristic properties. Depending on the mode of alignment of mesogens in LCEs, they were classified as nematic LCEs, smectic LCEs, cholesteric LCEs, *etc.* [9].

**Figure 4:**
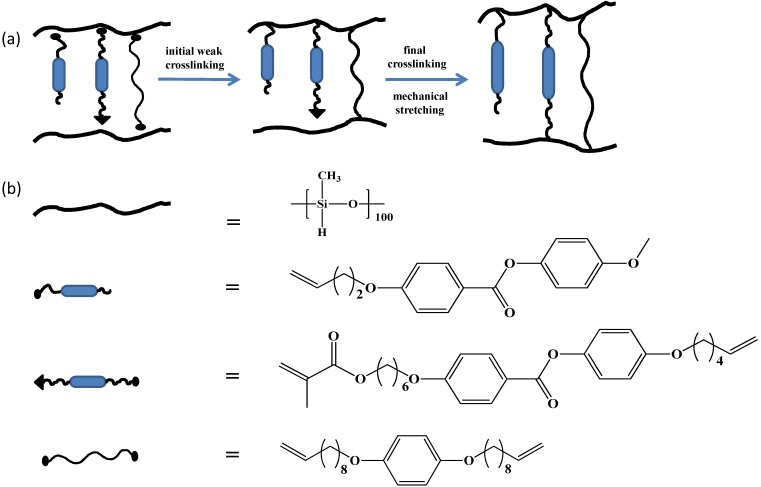
(**a**) Schematic representation of synthetic strategy proposed by Küpfer and Finkelmann, for the synthesis of monodomain nematic Liquid-Crystalline Elastomers (LCEs); (**b**) chemical structures of polymer chain, LC monomer and crosslinkers used for the synthesis monodomain nematic LCEs [29].

The concept of LCEs was first proposed by de Gennes [28], since then it has been investigated extensively by researchers. A major breakthrough in the area of LCEs took place when Küpfer and Finkelmann [29] successfully synthesized nematic monodomain LCEs (see Figure 4). Since then, a variety of LCEs with various structures of backbone chain, along with various kinds of mesogens, have been prepared. One of the important factors in the synthesis of LCEs is to perform the polymerization reaction at temperatures where the system exhibits a LC Phase. LCEs are synthesized by several synthetic routes. One can distinguish them into four different synthetic pathways.

The first method is commonly used and utilizes siloxane chemistry. In this synthetic route linear, non-functional/functional polyhydrosiloxane chain is coupled with the mesogenic groups and a crosslinking agent in one step. This reaction is platinum catalyzed, which results in the attachment of mesogens and crosslinking moieties to polyhydrosiloxane backbone [30]. Due to the reaction kinetics, vinyl groups react approximately two orders of magnitude faster than methacryloyl groups. This results in the crosslinking to occur in two steps. Fast reaction of vinyl groups leads to a weakly crosslinked network. Complete crosslinking is achieved in the second step by slowly reacting methacryloyl groups at high temperature. By using this synthetic method, various types of LCEs, namely end-on mesogens [29], side-on mesogens [31,32], photosensitive side-groups [33] and main-chain polymers [34] have been produced. The benefit of this chemical route is that it is easy to perform and mesogenic compounds can be exchanged without making considerable changes in the system. The problem with the method is that resulting networks are difficult to purify. Due to an incomplete reaction, low molar mass material (unreacted mesogens or crosslinker) can remain in the elastomer, which might migrate and phase separate. The only method for removing these impurities is to extract them with a suitable solvent from the elastomer, which is time consuming and is not always complete [9].

**Figure 5:**
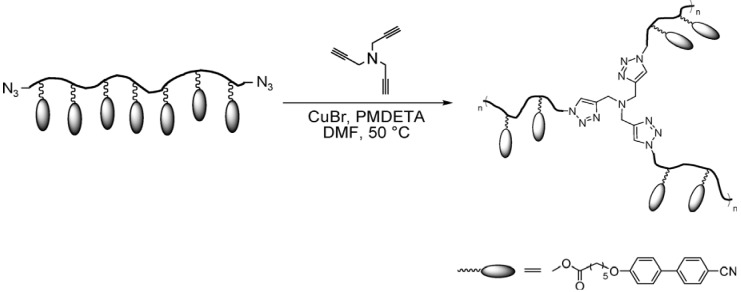
Crosslinking of telechelic polymer by reacting triacetylene species with azide functionalized LC polymer by “click chemistry”. Reprinted with permission from [36]. Copyright 2008, American Chemical Society.

The second synthesis strategy is also a two-step method where initially liquid-crystalline polymer is synthesized, which contains additional functional groups. These functional polymers are then mixed with a multifunctional crosslinking agent that reacts selectively with the functional groups, which results in network formation. This strategy has been widely used for the crosslinking of polyacrylate or methacrylates. The crosslinking can be done by coupling of isocyanates to alcohols [35], “click” reaction of azides and acetylenes [36] (see Figure 5), reaction of active ester and amines [37] and by hydrosilylation reaction [38]. The purification of the product is comparatively easier than the first synthesis route as LC polymers can be purified before the final crosslinking step.

**Figure 6:**
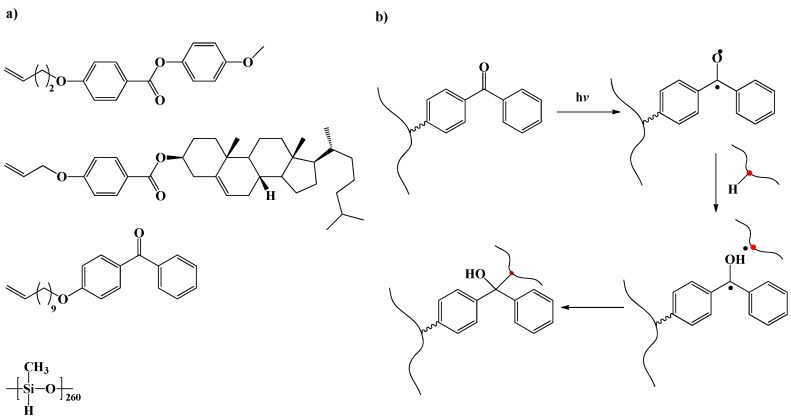
(**a**) Chemical structures of the molecules utilized for the synthesis of photo-crosslinked monodomain LCEs by Komp *et al.* [40]; (**b**) schematic representation of photoinduced crosslinking mechanism.

In the above mentioned two synthetic concepts, to achieve monodomain LCE is challenging as it requires the use of solvent during the crosslinking step to ensure miscibility of the reactants. The use of solvent leads to a mixture which have some parts in an isotropic state and that makes the monodomain orientation of mesogens difficult [9]. In the third route, LC polymer contains crosslinkable groups that can be crosslinked photonically [39,40,41,42] (see Figure 6) or by thermal/UV initiation [43,44,45]. The disadvantage of this synthetic route is the difficulty in achieving high degrees of crosslinking as one uses LC prepolymer that contains crosslinkable groups and steric hindrance, which makes it difficult to achieve high degrees of crosslinking. By this approach high purity can be achieved (same as second synthetic route) as LC polymer can be purified before the crosslinking step.

**Figure 7:**
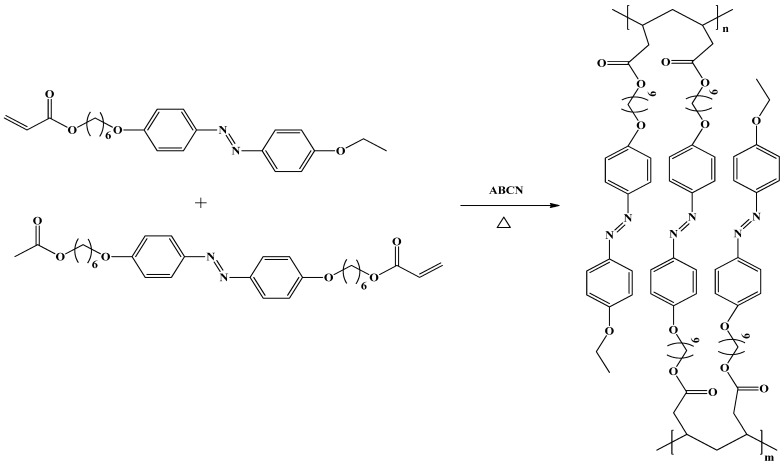
Synthetic route for the synthesis of photosensitive LCEs [49]. The polymerizations were initiated thermally by mixing monomer and crosslinker together with the initiator.

A fourth synthetic pathway uses a completely different approach. LCEs are prepared in one step by mixing LC monomer, radical initiator and multifunctional crosslinker together (see Figure 7). The polymerization reaction to yield LCE could be done thermally or with UV irradiation, depending on the type of initiator used. By using this approach, several types of LCE have been synthesized. For instance, acrylate functionalized monomer mixed together with radical initiator (thermal/UV), using diacrylate as crosslinker, is employed to yield LCE [46,47,48,49]. Main chain LCEs, which utilize this synthetic approach, have been synthesized by polymerizing LC monomer (with vinyl and mercapto group) and multifunctional crosslinker (with two vinyl and mercapto groups) using a radical photo-initiator [50,51,52].

## 4. Azobenzene Chromophore

**Figure 8:**
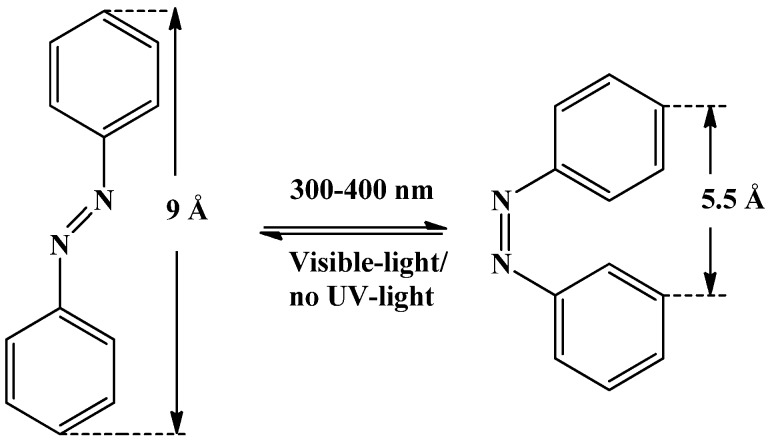
*Trans-cis* isomerization of azobenzene.

Photo-responsive smart polymers can be synthesized by functionalizing the material with photosensitive molecules such as cinnamic acid (CA), cinnamylidene or azo compounds. Out of these azobenzene is the most widely used photosensitive molecule due to its fast response on exposure to appropriate wavelength of light [18]. Azobenzene is composed of two aromatic rings where an azo linkage (−N=N−) joins the two phenyl rings. Different type of azo compounds can be obtained by substituting an aromatic ring with various substituents to change geometry and electron donating/withdrawing mechanism. Members of this class of chromophores share numerous spectroscopic and photo-physical properties; however, it is useful to consider them generally based on their photochemistry and in particular, the *π*-conjugated system which gives strong electronic absorption in the UV and/or visible portions of the spectrum depending on the ring-substitution pattern. The azobenzene molecule is quite rigid and exhibits LC behavior, which makes them useful candidate for the synthesis of photo-responsive LC materials. One of the interesting properties of azobenzene and its derivatives is its fast and reversible photoisomerization, which takes place upon irradiation with suitable wavelength of light (Figure 8). Azobenzenes possess two isomeric configration: a thermally stable *trans* state and a meta-stable *cis* form. Under UV irradiation, the *trans* azobenzenes will be efficiently converted to the *cis* form, which will reduce the molecular size (the distance between 4 and 4’ carbons decreases from 9 Å to 5.5 Å) [53]. This *cis* form will thermally revert to the more stable *trans* form (rate is determined by the molecule’s particular substitution pattern) as the light source is switched off or switching back by illumination with visible light. This extremely clean photochemistry gives rise to the numerous remarkable photo-switching and photo-responsive behaviors observed in these systems [4,54,55,56,57].

## 5. Preparation of Oriented Liquid-Crystalline Network (LCN) Films

Oriented LCN films are generally prepared either (i) by a mechanical stretching of a weakly crosslinked network which unfolds the chains and final crosslinking step (under load) would fix them in a LC state or (ii) by performing a polymerization reaction between alignment layer that provides an anchoring action for LC molecules.

**Figure 9:**
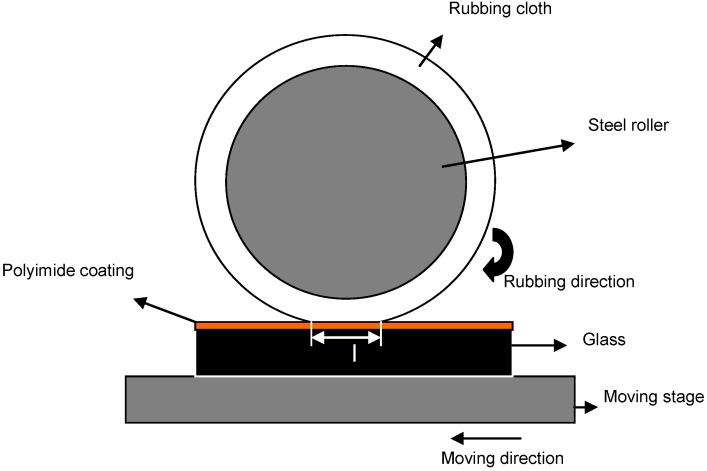
Schematic representation of experimental setup for fabrication alignment layers [60].

One of the requirements for the mechanical stretching route is that the material should be able to withstand the mechanical force applied during stretching operation. The preferred method for obtaining oriented LCN films is to achieve orientation by performing polymerization reaction between rubbed polyimide alignment layers. Polyimides are generally preferred as an alignment layer because of their excellent properties with respect to chemical resistance: thermal stability, adhesion to substrates, transparency and high resistivity [58,59]. Polyimide films are rubbed mechanically by using a mechanical roller, which is coated with nylon or rayon, shown schematically in Figure 9. The polymerization reaction is carried out between the rubbed polyimide layers, which results in oriented LC films. The drawback of this method is that orientation of molecules only takes place near the surface, which means that it is difficult to obtain orientation in thicker films.

Recently Yu and coworkers have reported a method to prepare highly oriented photo-deformable crosslinked liquid-crystalline polymer/carbon nanotube (CLCP/CNT) nano composite films [61]. The composite films were prepared by performing a polymerization reaction between glass cell that is covered with CNT sheet and also the CNT sheet in-between. They found that aligned nanostructure of CNT effectively orients the azobenzene containing CLCP along the length of CNTs through a facile melting process without employing any other aligning layer. The resulting oriented nanocomposite films exhibit bending and unbending behavior on irradiation with UV and visible light. Additionally, the incorporation of CNT sheets remarkably increases the mechanical strength and electrical conductivity of photo-responsive CLCP films.

## 6. Light Responsive LCNs

### 6.1. Photosensitive Shape-Changing LCEs & LCNs

**Figure 10:**
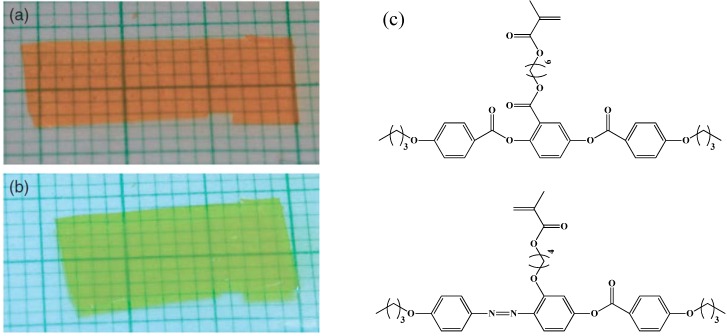
Side-on photo-responsive Liquid-Crystalline Network **(**LCN) film, (**a**) before UV irradiation; (**b**) film contraction under UV irradiation; (**c**) chemical structures of side-on LC monomers used for the synthesis of LCE. Reprinted with permission [63]. Copyright Wiley-VCH 2003.

Photo-responsive shape-changing LCNs have attracted researchers’ interest due to their capability to selectively alter their shape in response to changes in the stimulus. The advantage photosensitive LCNs offer over amorphous smart polymers is anisotropy. The amorphous photosensitive smart polymers do not exhibit microscopic or macroscopic order, which results in the photomechanical deformations in an isotropic and uniform way *i.e.*, with no preferential direction for deformation. The first example of amorphous photosensitive polymer was synthesized by Eisenbach [62] (see Figure 10). They demonstrated approx. 0.2% contraction of the film on UV irradiation and expansion back to original position over irradiation with visible light. This contraction is a result of *trans-cis* isomerization of azobenzene chromophores, which were reversed due to *cis-trans* transformation by the visible light. Unlike amorphous photosensitive polymers, light responsive LCNs offers fast and large deformation in preferential direction *i.e.*, in the alignment direction of chains. For this reason, LCNs functionalized with photosensitive molecules are being produced, which have properties of both LCs and elastomers. This was demonstrated initially by Finkelmann and coworkers [33], where they succeeded in inducing a contraction by 20% in an azobenzene containing monodomain LCE upon exposure to UV light to cause the *trans-cis* isomerization of the azobenzene moiety. *Trans-cis* isomerization of azobenzene moieties resulted in the lowering of nematic order, which causes a significant uniaxial deformation of the LCs along with the director axis. Although the uniaxial deformation demonstrated by Finkelmann and coworkers was significant, the rather slow response (from minutes to several hours) over irradiation with light was a major obstacle in finding some interesting applications. This slow system response was later on rectified by Li *et al*. [63]. By synthesizing side-on nematic photosensitive LCEs, they were able to reach 12–18% contraction of the film in approx. 1 min over irradiation with UV light.

Terentjev and co-workers [64,65] studied the effect of different compositions and crosslinking configuration on the uniaxial contraction behavior of these side-on LCNs. They found that the magnitude of photomechanical deformation is governed by proportion and position of azobenzene moieties.

### 6.2. Photoinduced 3-D Movements of LCNs

**Figure 11:**
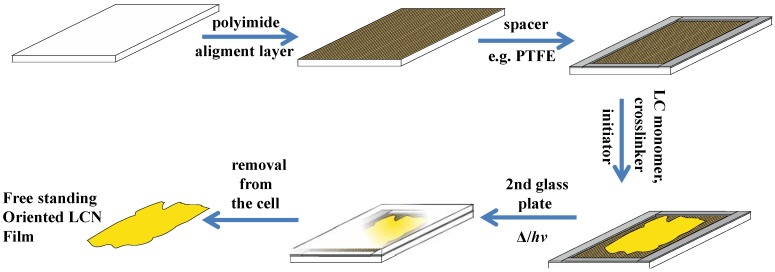
Schematic representing the fabrication procedure for synthesis of oriented LCE films. Initially the glass slabs are spin coated with polyimide and baked, followed by rubbing of these films to yield alignment layers. Then monomer, crosslinker and initiator is sandwiched between the coated glass plates and polymerized using heat/UV. The resulting film is then removed from the plates.

The motivation for the development of multi-dimensional photo-responsive LCNs stems from the need to broaden the potential application areas especially as robotic arms or motors. Ikeda *et al*. were first to demonstrate the photoinduced bending in azobenzene containing LC gels [47] and LCEs [47,53,66]. They prepared crosslinked films by in-situ photoinduced polymerization of azobenzene based LC monomer and diacrylate as a crosslinker between glass slabs, coated with rubbed polyimide films to yield oriented films, shown schematically in Figure 11. These films showed anisotropic swelling in good solvents such as toluene or chloroform, but no or very little swelling in poor solvents such as ethanol. Upon exposure of these films in toluene to UV light (360 nm), the film bends towards the irradiation direction due to *trans-cis* isomerization of azobenzene moieties. Complete bending was achieved approximately in 20 s, while exposure to visible light (450 nm), causes the unbending of the film, which was a caused by *cis-trans* back isomerization of azobenzene molecules.

**Figure 12:**
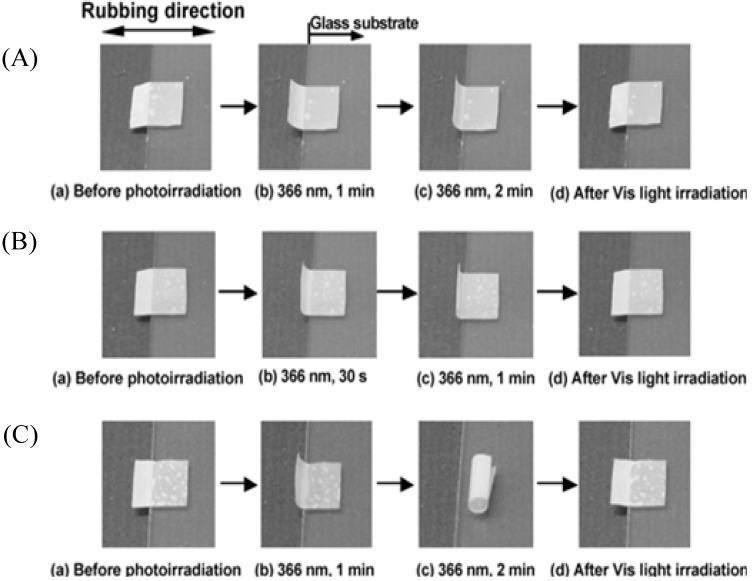
Photoinduced bending and unbending behavior of photo-responsive LCEs with different crosslinking densities. (**A**) LCE with 5% crosslinker; (**B**) 10% crosslinker; (**C**) 50% crosslinker. Adopted from [66]. Copyright (2004) American Chemical Society.

The same bending and unbending of these films were observed in air, when the films were first heated over its glass transition temperature (*T*_g_) and then irradiated with UV/visible light to cause bending/unbending of LCE films. When the film was rotated by 90°, the bending was still observed in the rubbing direction, which expresses that bending is anisotropic. Schematic representation of anisotropic bending/unbending mechanism of oriented azobenzene based LCNs is demonstrated in Figure 2. Initially, the film is heated above its *T*_g_ to give freedom to polymer segments so that chains have enough flexibility to move. On irradiation of the crosslinked film with UV light, 99% of incident photons are absorbed by azobenzene molecules at the surface (<1 µm) due to the high absorption coefficient of azobenzene molecules. This results in *trans-cis* isomerization of azobenzene moieties at the surface while the rest of azo molecules in the bulk remain unchanged. That leads to a volume contraction taking place only at the surface of LCE film. Therefore, bending is induced towards the irradiation direction. Moreover, since azobenzene chromophores are aligned only in one direction (*i.e.*, along the rubbing direction) the films could only change their shape in one direction on *trans-cis* photo-isomerization [47]. Ikeda and coworkers also analyzed the effect of crosslinking density on the bending behavior of monodomain crosslinked LC films [66] (see Figure 12). They reported that the films with higher crosslinking densities showed higher bending extents. Higher amount of crosslinker results in greater alignment of azobenzene moieties along the rubbing direction. Therefore, exposure to UV light results in the volume contraction along the rubbing direction and hence, overall bending extent of photosensitive LCE film is boosted.

**Figure 13:**
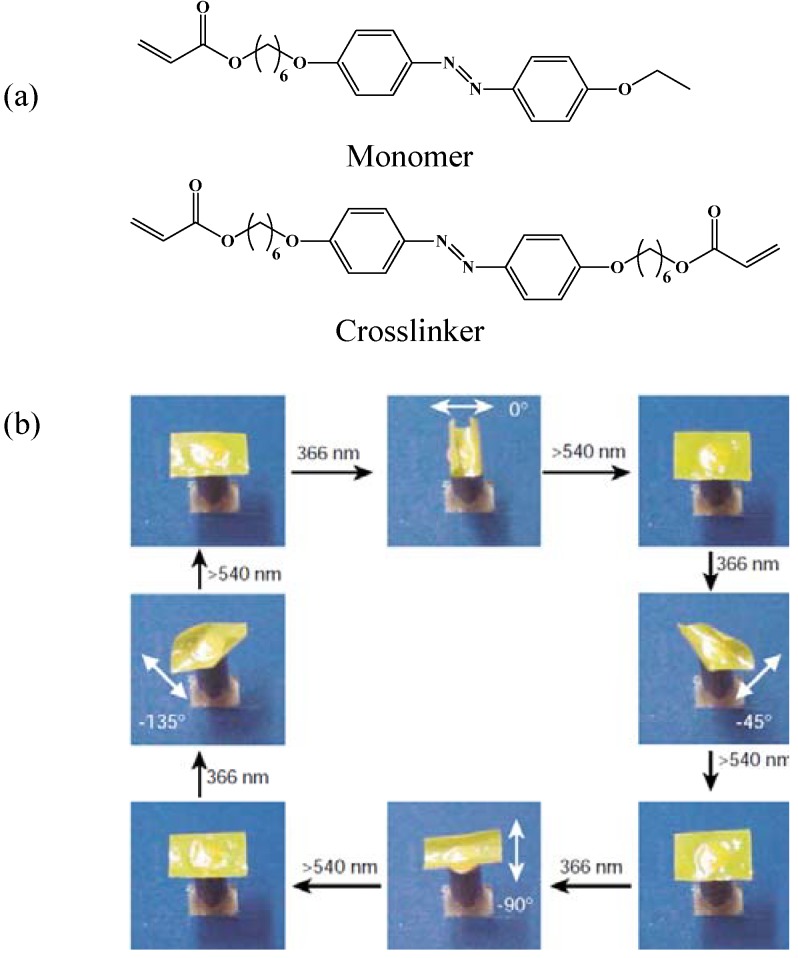
(**a**) Chemical structure of monomer and crosslinker used in the synthesis of LCN films. (**b**) Photographs of direction controlled bending of polydomain LCN film by irradiation with linearly polarized UV light (366 nm) at different angles of polarization. The bent films reverted back to initial flat state by irradiation with visible light (540 nm). Reprinted with permission from Macmillan Publishers Ltd. [Nature] [53], copyright (2003).

By synthesizing polydomain azobenzene based LCEs and using selective absorption of linearly polarized UV light, it was demonstrated [53] (see Figure 13) that one could selectively alter the bending direction of the film by changing the polarization direction of incident light. The films bend significantly, in parallel to the irradiation direction of light.

**Figure 14:**
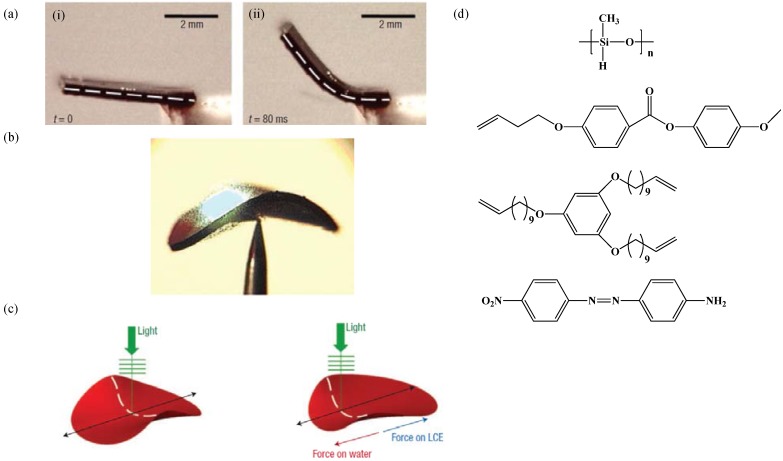
(**a**) Photo-mechanical response of the dye-doped LCE film, (i) before irradiation with 514 nm green laser, (ii) after illumination the sample bends about 45°. (**b**) The shape deformation of the dye-doped LCE sample on irradiation with 514 nm light. (**c**) Schematic representation of the mechanism underlying the locomotion of the LCE sample. (**d**) Chemical structures molecules employed in synthesis of dye doped nematic LCE. Reprinted with permission from Macmillan Publishers Ltd [Nature Materials] [67], copyright (2004).

Shortly after, Muhoray and co-workers [67] demonstrated that by dissolving azobenzene dyes into an LCE system, the mechanical deformation can be greatly enhanced (more than 60° bending) on non-uniform illumination with visible light. When this dye-doped sample was placed over a water surface and irradiated, LCE swims away from light, the action resembles that of flatfish (Figure 14).

White and coworkers explored the photoinduced bending behavior of LCE film on exposure to single laser beam at room temperature [68]. The response to laser light was extremely fast (approx. 170°/s) and bi-directional bending was achieved by simply changing the polarization of the beam in orthogonal directions. The similar room temperature bending and twisting of LCN films was achieved by synthesizing densely crosslinked polymer networks that contains azobenzene moieties in twisting configuration [69]. The chirality of the twisted configuration has been exploited to produce a coiling motion of the film over irradiation with UV light.

The effect of initial alignment of mesogens on photoinduced bending behavior of LCE films was studied [70]. When exposed to UV light, it was also found that LC mesogens aligned homotropically in LCE and resulted in bending away from the direction of light source. Azobenzene mesogens are aligned perpendicularly to the film surface in homeotropic film, thus exposure to UV light induces isotropic expansion, which leads to bending of the film away from the light source. The effect of degree of order of mesogens was studied with the use of ferroelectric LCEs, where the degree of alignment could be triggered with an electric field [71]. The photoinduced mechanical stress generated by this system was about 220 KPa, which is quite similar to the contraction stress of human muscles (~300 KPa).

A high frequency photo-driven deformation in azobenzene based monodomain or polydomain LCNs, due to *trans-cis-trans* back isomerization, was demonstrated by Bunning *et al*. [72,73,74,75,76]. Subjecting monodomain azobenzene LCNs to higher wavelength argon-ion laser light of 457–514 nm causes bending of the cantilever towards the laser source and blockage of laser beam results in recovery of the cantilever to its original position, due to elastic recovery of unexposed regions and entropic restoring forces governed by the network within the exposed region, rapidly reorienting the photo-driven mesogens to realign back to monodomain configuration [75]. They also synthesized UV pretreated azobenzene based LCN of polydomain orientation to have two 1 µm thick *cis* isomer rich layers. On exposure of Ar+ laser from one side, the film bends away from the laser due to *cis-trans* isomerization. The *cis-trans* isomerization at the exposed surface restores the LC order at the surface, which removes the contractile strain on the front surface relative to the bulk [72]. Later on, as the concentration of *trans* isomers increases, the *trans-cis* isomerization occurs, which results in bending toward the light source. They also have demonstrated the photomechanical response of glassy azobenzene polyimide networks on exposure to linearly polarized 442 nm light. It was shown that increasing the crosslinker concentration results in enhancement of bending extent of cantilever from 5° to 20° [77].

**Figure 15:**
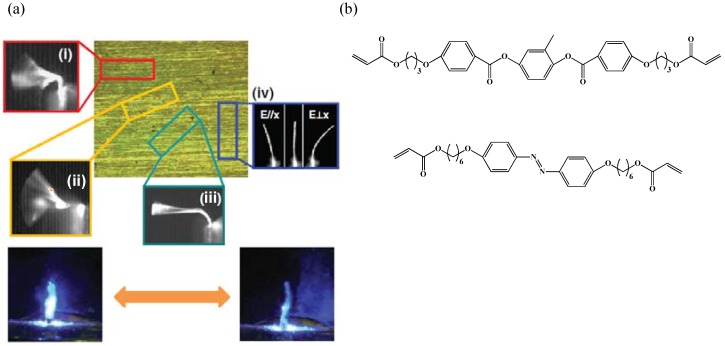
(**a**) Polarization optical micrograph of azobenzene containing LCN and photomechanical response of LCN films with nematic director cut at (i) 0°, (ii) 15°, (iii) 45°, (iv) 90° to the long axis of the LCN film. (**b**) Chemical structure of molecules used for the synthesis of photo-responsive LCN cantilevers. Reprinted with permission [78]. Copyright Wiley-VCH 2011.

Recently, high frequency and large amplitude flexural torsional oscillations have been demonstrated in monodomain azobenzene based LCN cantilevers having comparatively lower concentrations of azobenzene chromophores [78] (see Figure 15). Flexural torsional oscillations in these monodomain azo LCN were realized by aligning the nematic director at intermediate angles to the long axis of the cantilever. On exposing LCNs to light from a 442 nm coherent wave laser resulted in non-uniform strain through the sample thickness resulting in bending, while adjustment in the orientation of the nematic director creates a shear gradient that causes twisting of the cantilever. These flexural torsional oscillations imitate the flapping motions of insects that undergo 3-D movements (bend, twist and sweep) to fly [79].

**Figure 16:**
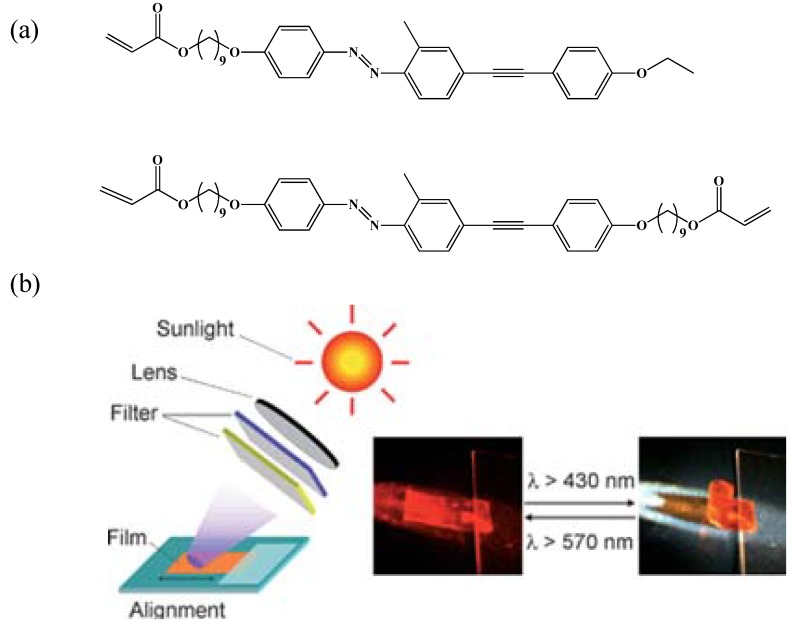
(**a**) Chemical structure of LC monomer and crosslinker used to synthesize an azotolane LCN film, (**b**) photo-induced bending and unbending behavior of the CLCP films on exposure to sunlight through a lens and glass filters to let desired wavelength of light pass through. Over irradiation with >430 nm sunlight the film bends towards the light and on exposing the CLCP films to >570 nm light results in unbending of the films to initial flat position. Reproduced from [80] with permission of The Royal Society of Chemistry.

Yu *et al*. successfully demonstrated that photoinduced bending and unbending in crosslinked LC films could be achieved over irradiation with visible light by incorporating azotolane chromophores in the side chain [80] (see Figure 16). This is due to longer conjugated structure of azotolane groups in the side chain, which results in the maximum absorption occurring at higher wavelength (*i.e.*, 384 nm). On irradiation of crosslinked azotolane LC films with visible light at 436 nm results in bending towards the acitinic light due to *trans-cis* isomerization of azotolane groups. Moreover, irradiation with visible light at 577 nm results in unbending of the films due to *cis-trans* back isomerization.

Kondo *et al.* have reported that the photo-mobil properties of photo-active LCE films can be altered by the concentration and location of azobenzene molecules [81]. They found that higher bending in photosensitive films could be achieved by decreasing the feed ratio of azobenzene moieties in LCE film. As the feed ratio of azobenzene molecules decreases, penetration depth and degree of isomerization is enhanced, this results in enhanced photoinduced movements. Moreover, it was found that azobenzene chromophores at the crosslink are more effective in photoinduced bending process than those in the side chains.

More recently, precisely controlled three dimensional photo-mobility of crosslinked azobenzene LCP have been reported by Ikeda and coworkers [82]. The principle of photoinduced bending and unbending of these crosslinked fibers were same as reported for azobenzene containing LCEs [47], where bending was achieved by irradiation with 366 nm actinic light and reversal to original shape by exposing to visible light having wavelength of >540 nm.

## 7. Light Activated Shape-Memory LCNs

**Figure 17:**
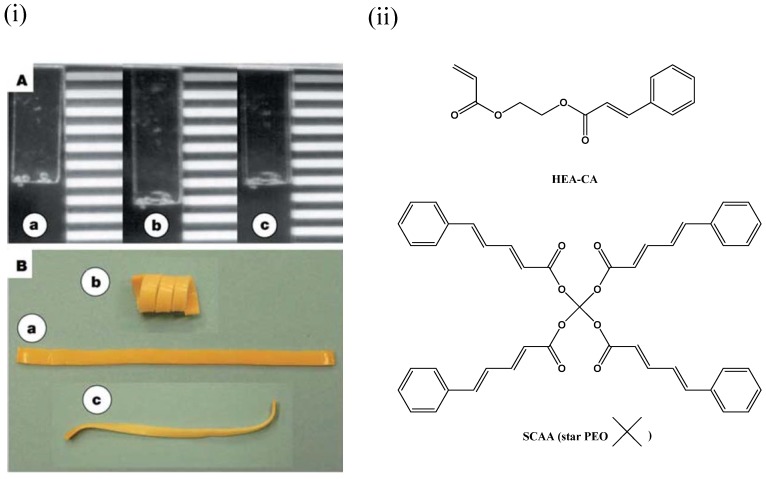
(**i**) Shape-memory effect of photo-responsive polymer demonstrated by Lendlein *et al*. [13]. (**A**) grafted polymer film, (a) Permanent non-elongated shape, (b) temporary shape after mechanical elongation and irradiation with light >260 nm, the film stayed in the elongated state after removal of stress, (c) recovered shape, after irradiation with light <260 nm. (**B**) An interpenetrating polymer network film, (a) permanent shape, (b) temporary cork spiral shape, which results after irradiation from one side, (c) recovered shape after irradiation with UV light of wavelength <260 nm. Reprinted with permission from Macmillan Publishers Ltd [Nature] [13], copyright (2005).

Light is a very useful trigger to alter the shape of polymers but there are very few publications that address light activated shape-memory polymers [13,83,84,85]. The pioneering work in this area was demonstrated by Lendlein *et al*. [13] (shown in Figure 17), where they used polymers, functionalized with cinnamic groups to exhibit photoinduced shape-memory effect. Initially, the photo-responsive polymer film was stretched by applying mechanical force and then exposed to UV light at wavelength higher than 260 nm to fix the elongated shape due to photo-induced [2+2] cycloaddition reaction. The original shape was recovered by exposing the films to wavelength of light shorter than 260 nm at room temperature, which causes the decrosslinking of photosensitive netpoints. In addition, when only the top side of the mechanically stretched film was irradiated with UV light (>260 nm), a cork screw spiral shape was obtained as the stress was released. The spiral shape was obtained due to the formation of two layers, *i.e.*, the top layer is well fixed due to formation of net points and the bottom layer keeps its elasticity. The work of Havens *et al*. [83] also uses the similar photo crosslinking strategy to photo fix the shape and decrosslinking by irradiation with different wavelength of light.

**Figure 18:**
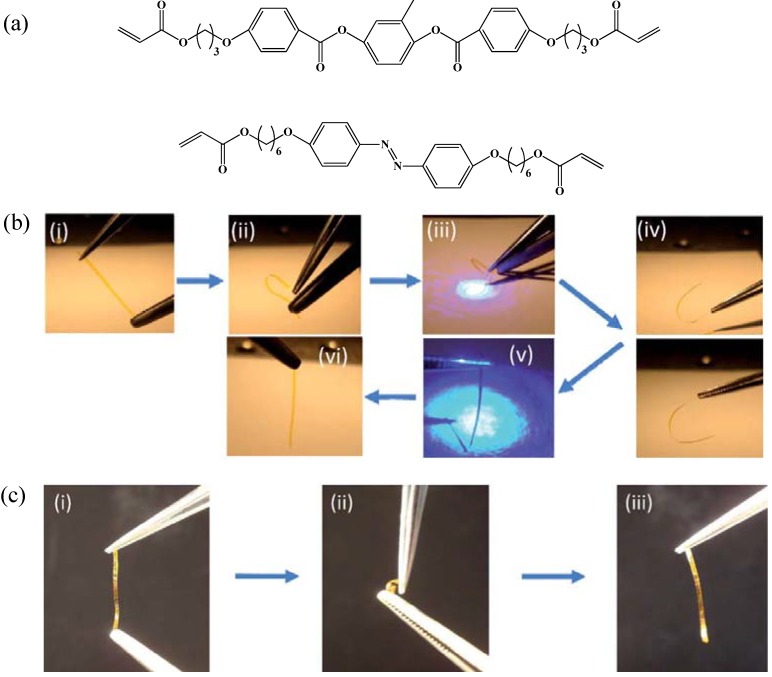
(**a**) Chemical structure of molecules used in synthesizing light-activated shape-memory LCNs. (**b**) Light activated shape-memory of free standing LCN films, (i) permanent shape, (ii) initial temporary shape by mechanical deformation, (iii) photo-fixing of the temporary shape by exposing to 442 nm light, (iv) shape retention after turning off the light, (v) shape recovery by exposing the LCN films to right-handed circularly polarized light, (vi) complete recovery of free standing film to permanent position. (**c**) Behavior of freestanding films without photo-fixing, (i) permanent shape, (ii) temporary shape by mechanical deformation, (iii) without photo-fixing the mechanically deformed shape restores to initial flat shape on removal of mechanical force. Reproduced from ref [86] with permission of The Royal Society of Chemistry.

Recently, White and coworkers have showed that one can use azobenzene based LCP networks to synthesize light activated shape-memory LCN [86] (see Figure 18). Azobenzene based LCN networks were synthesized by photoinduced polymerization of acrylate based monomer and azobenzene containing crosslinker. Monodomain LCN networks were synthesized by performing polymerization at 75 °C, while polymerization at 125 °C results in polydomain samples. Initially, the films (both monodomain and polydomain) were deformed at 100 °C (well above their *T*_g_) and then quenched to room temperature to thermally fix the hook-like temporary shapes. Later on, the films were exposed to linearly polarized 442 nm laser light which causes the bending of the film. On removal of 442 nm light the film retains its bent shape. This photo-fixed shape is due to light directed rearrangements to the polymer chains in the glassy matrix (analogous to thermal fixing of glassy shape-memory polymer) [86]. By exposing the films to 442 nm circularly polarized light undoes the photo-fixing, restoring the mechanically deformed hook-like shape. The permanent shape of the LCN was achieved by heating the films over their *T*_g_, which causes the relaxation of the stretched polymer network chains to the thermodynamically more stable configuration. Lee *et al*. demonstrated that the photomechanical response of glassy, azobenzene-functionalized polyimides can be tailored by manipulating the energy state of the glass via physical aging [85]. They reported that physical aging of azobenzene functionalized polyimides increases the density of glass, reduces the local free volume and thus reduces the minima in local conformation space. These factors influence the magnitude of macroscopic strain and the ability of material to shift from shape fixing to shape recovery, respectively.

## 8. Some Applications of Photo-Responsive LCNs

**Figure 19:**
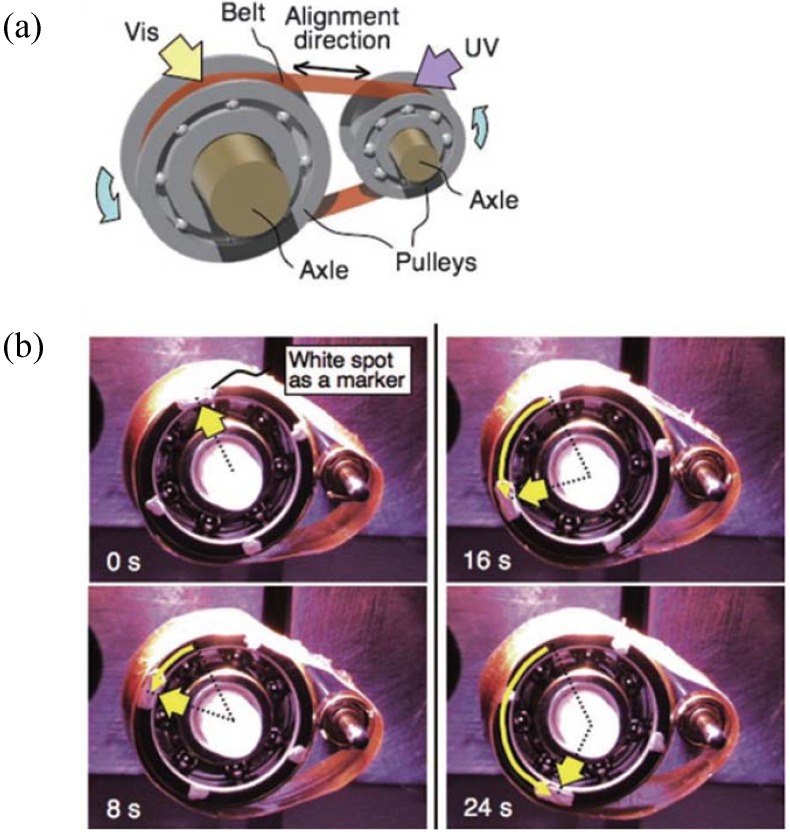
A photo-driven plastic motor with the laminated LCN film. (**a**) Schematic representation of the photo-driven plastic motor setup, (**b**) photographs of motor operation, upon irradiation with UV light on one side, the film contracts and irradiation with visible light from the other results in expansion, which results in the rotation of motor. Reprinted with permission [87]. Copyright Wiley-VCH (2008).

For almost a decade, material scientists have tried to develop artificial actuators by synthesizing photo-responsive LCN. As mentioned in the previous sections, light is an interesting stimulus to precisely trigger the motion of smart materials as it allows remote actuation without employing any mechanical or electrical forces. This makes photoactive LCN a potential candidate for various novel applications in engineering and medicine. Ikeda and coworkers have demonstrated that plastic motor can be driven by the sophisticated motion of photo-responsive LCE films laminated on polyethylene (PE) sheet over irradiation with light [87]. They prepared a plastic belt by connecting the two ends of laminated LCE/PE film and then placed on a pulley system as illustrated in Figure 19. Exposing the belt to UV light from the top and visible light from the bottom simultaneously, induces the rotation of the belt which finally drives the pulleys. By using the same material system *i.e.*, laminated LCE/PE film, they showed 3-D movements such as an inchworm walk and flexible robotic arm motion [88].

**Figure 20:**
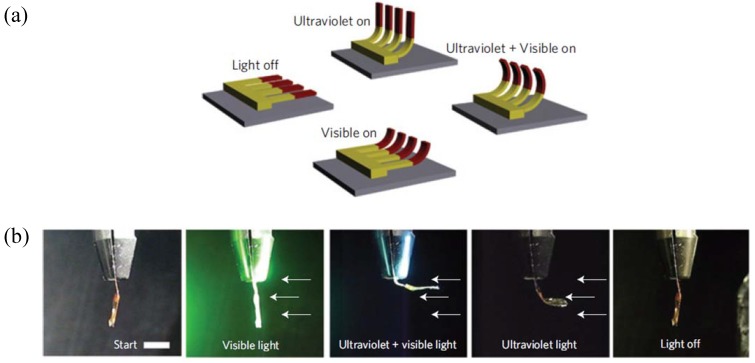
(**a**) Schematic representation of photo-driven artificial cilia by exposing to controlled wavelength of light. (**b**) Response of LCN actuator to two different wavelengths of light. Reprinted with permission from Macmillan Publishers Ltd. [Nature Materials], copyright (2009) [48].

Broer and coworkers synthesized photo sensitive micro-actuators from LCN by inkjet printing in combination with the self-organizing LC mesogens, shown in Figure 20. These micro-actuators mimic the motion of artificial cilia. They found that upon irradiating the sample with light the sample bends away from the light source. They were able to control the bending modes by exposing the LCN to an appropriate wavelength of light [48].

Recently Yu and coworkers have demonstrated the use of photo-sensitive LCN films to activate membrane pumps [89] and as a valve membrane [90]. In addition, they also developed a micro-robot made out of a laminate of photo responsive LCN film and stretched PE sheet, which could move a weight up to 10 mg [91].

## 9. Conclusions

Photo-responsive LCNs have been explored extensively in past decades as they can remotely and precisely trigger the shape that makes them a potential candidate in various applications such as soft actuators and photo-driven sensors. Photo-responsive shape-changing LCNs have been utilized by material scientists to mimic various 2-D and 3-D movements, while on the other hand there is substantial potential in the area of photo induced shape-memory LCN, as there were only few works reported.

In this review, we provided a summary of recent developments in the area of photo-responsive shape-memory and shape-changing liquid-crystalline polymer networks. As research in this area is quite scarce, still many challenges and opportunities lie ahead of us. For instance, the mechanical forces generated by these photo-responsive LCNs are quite low and the efficiency of photo energy conversion is also not optimal. Looking into future, we anticipate major advances in the area of photo-responsive SMPs and also some “real life” applications for photo-responsive LCNs as actuators, muscles and sensors.
